# Dihydroberkleasmin A: A New Eremophilane Sesquiterpenoid from the Fermentation Broth of the Plant Endophytic Fungus *Pestalotiopsis photiniae*

**DOI:** 10.3390/molecules16021910

**Published:** 2011-02-23

**Authors:** Xiao-Long Yang, Su Zhang, Hua-Jie Zhu, Du-Qiang Luo

**Affiliations:** 1 Key Laboratory of Pharmaceutical Chemistry and Molecular Diagnosis of Ministry of Education, Hebei University, Baoding 071002, China; 2 College of Pharmaceutical Science, Hebei University, Baoding 071002, China; 3 College of Life Science, Hebei University, Baoding 071002, China

**Keywords:** *Pestalotiopsis photiniae*, eremophilane sesquiterpenoid, dihydroberkleasmin A

## Abstract

Dihydroberkleasmin A (**1**), a new ester-substituted sesquiterpenoid related to the eremophilane class, together with the known compound berkleasmin C (**2**), were isolated from the fermentation broth of the plant endophytic fungus *Pestalotiopsis photiniae*. The structure of dihydroberkleasmin A (**1**) was elucidated by extensive spectroscopic analysis. The stereochemistry was assigned by comparison of the NMR spectroscopic data with those of berkleasmin A.

## 1. Introduction

Fungi of the genus *Pestalotiopsis* (Amphisphaeriaceae), as one class of the most widely distributed endophytic fungi, are common in their distribution, and many are saprobes, while others are either pathogenic or endophytic to living plants [1,2,3,4]. Since discovery of the anticancer agent taxol from an endophytic fungal strain of the genus *Pestalotiopsis* [5,6], interest in searching for bioactive compounds from this fungal genus has increased considerably. Up to date, about 300 species of the genus *Pestalotiopsis* have been recorded in China, but only about 10% of these species referred to chemical investigations. Previous chemical studies of some species of this genus have afforded a variety of bioactive metabolites [7,8,9,10,11,12,13,14,15,16,17,18]. In the course of our research on bioactive metabolites of the genus *Pestalotiopsis* in China, the present study was undertaken to investigate the chemical constituents of the culture broth of *Pestalotiopsis photiniae* isolated from the branch of *Podocarpus macrophyllus* in Hainan (People’s Republic of China), and have led to the isolation of a new eremophilane sesquiterpenoid named dihydroberkleasmin A (**1**) and one known compound, berkleasmin C (**2**). Details of the isolation and structural elucidation of **1** are reported herein.

**Figure 1:**
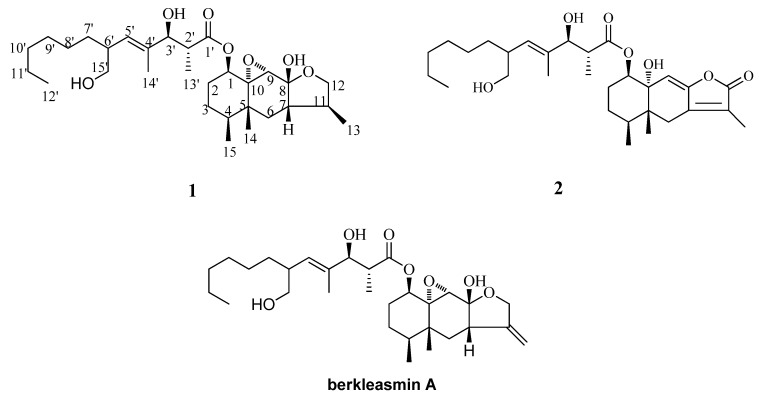
The structures of compounds **1**, **2** and berkleasmin A.

## 2. Results and Discussion

Compound **1** was obtained as an optically active white powder, [α]_D_^2^^2.0^ = +70° (c = 0.1, MeOH) that gave a quasi-molecular ion peak at [M+Na]^+^ *m/z* 545.3458 in the HR-ESI-MS (positive mode), consistent with a molecular formula of C_30_H_5__0_O_7_ (calcd. for C_30_H_50_O_7_Na, 545.3454), requiring six degrees of unsaturation. The IR spectrum revealed absorption bands of double bond (1,604 cm^−1^), hydroxyl (3,424 cm^−1^) and carbonyl (1,736 cm^−1^) groups. There were 30 signals observed in the ^13^C-NMR spectrum (Table 1). Analysis of the ^13^C-NMR, DEPT, and HSQC spectra revealed that **1** contained one carbonyl carbon, seven oxygenated carbons, two olefinic carbons, eight methylene carbons, five methine carbons, one quaternary carbon, and six methyl carbons. Analysis of the ^1^H-NMR spectrum (Table 1) indicated the presence of six methyl signals including one tertiary methyl [*δ_H_* 1.62 (s), 1.15 (s), 1.13 (d, *J* = 7.2 Hz), 1.05 (d, *J* = 6.6 Hz), 0.89 (d, *J* = 6.7 Hz), 0.87 (t, *J* = 7.2 Hz)], one olefinic proton signal [*δ_H_* 5.23 (d, *J* = 10.1 Hz)], two oxygenated methylene protons signals [*δ_H_* 3.95, 3.54 (m), 3.63, 3.34 (m)] and three oxygenated methine proton signals [*δ_H_* 4.50 (m), 4.09 (d, *J* = 7.4 Hz ), 3.27 (s)]. By careful analysis of NMR data, we found that the spectral data of **1** were similar to those of berkleasmin A recently reported from the saprobic fungus *Berkleasmium nigroapicale* [19], and this suggested that **1** has a tricyclic sesquiterpene core attached to a long-chain acid through an ester linkage. The distinct differences between **1** and berkleasmin A are: the chemical shifts value at C-11 and C-13 of **1** [*δ_C_* 42.7 (d, C-11), 16.0 (q, C-13)] are absent in berkleasmin A [*δ_C_* 151.2 (s, C-11), 104.3 (t, C-13)]. In addition, the chemical shifts value at C-7 (*δ_C_* 48.3) and C-12 (*δ_C_* 72.3) in **1** were shifted downfield compared to berkleasmin A [*δ_C_* 44.6 (s, C-7), 69.9 (t, C-12)] because of replacement of the exomethylene group in berkleasmin A by a methyl group in compound **1**. 

**Table 1:** ^1^H-(600 MHz) and ^13^C-NMR (150 MHz) data for **1** in CDCl_3_, and the literature data for berkleasmin A [19].

No.	*δ_H_*	*δ_C_*	No.	*δ_H_*	*δ_C_*
1	4.50 (m)	74.8 (d)	1'		175.3 (s)
2	1.86, 1.77 (m)	28.5 (t)	2'	2.71 (dq, 7.3, 7.4)	42.6 (d)
3	1.44, 1.76 (m)	25.7(t)	3'	4.09 (d, 7.4)	79.1 (d)
4	1.58 (m)	38.9 (d)	4'		137.5 (s)
5		36.2 (s)	5'	5.23 (d, 10.1)	130.8 (d)
6	α 1.30 (t, 13.0)β 1.64 (dd, 13.0, 6.8 )	37.2 (t)	6'	2.56 (m)	40.8 (d)
7	1.70 (m)	48.3 (d)	7'	1.09, 1.29 (m)	31.3 (t)
8		102.3 (s)	8'	1.21–1.28 (m)	27.2 (t)
9	3.27 (s)	62.4 (d)	9'	1.21–1.28 (m)	29.4 (t)
10		62.8 (s)	10'	1.21–1.28 (m)	31.8 (t)
11	1.80 (m)	42.7 (d)	11'	1.21–1.28 (m)	22.6 (t)
12	3.54, 3.95 (m)	72.3 (t)	12'	0.87 (t, 7.2)	14.1 (q)
13	1.05 (d, 6.6)	16.0 (q)	13'	1.13 (d, 7.2)	15.0 (q)
14	1.15 (s)	15.3 (q)	14'	1.62 (s)	12.2 (q)
15	0.89 (d,6.7)	15.1 (q)	15'	3.34, 3.63 (m)	66.5 (t)

**Figure 2:**
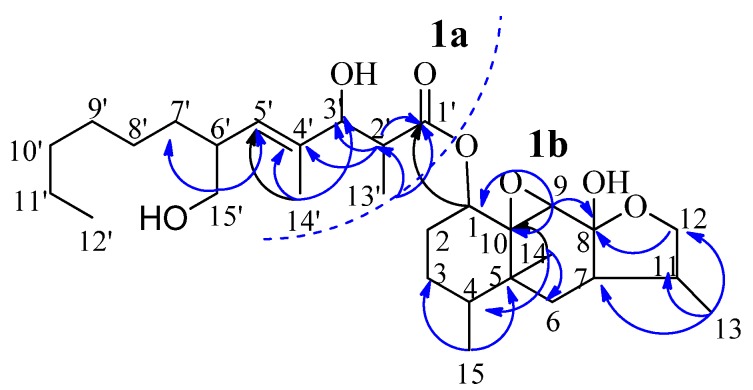
The fragments and selected HMBC correlations of **1**.

Further interpretation of the HMBC spectrum showed the following long-range correlations (Figure 2): from H-2' to C-1', C-3', C-4' and C-13', from H-3' to C-1', C-2', C-4', C-5', C-13' and C-14', from H-5' to C-3', C-14' and C-15', from H-6' to C-4', C-5', C-7' and C-15', from H_3_-13' to C-1', C-2' and C-3', from H-14' to C-3', C-4' and C-5', from H-15' to C-5' and C-7'.

**Figure 3:**
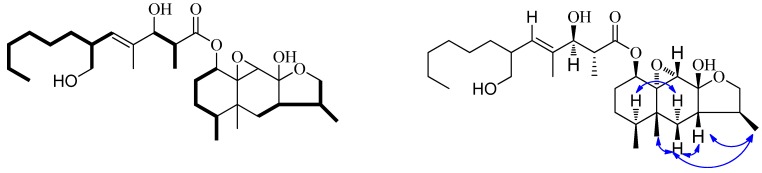
The ^1^H, ^1^H-COSY and key selected NOESY correlations of **1**.

The above spectral evidence, along with the proton spin system: H-3'/H-2' and H-2'/H_3_-13'; H-5'/H-6'/H-7'/H-8'/H-9'/H-10'/H-11'/H_3_-12' and H-6'/H-15' deduced from ^1^H, ^1^H-COSY (Figure 3) correlations, led to the establishment of the partial structure **1a** (Figure 2). In addition, HMBC spectrum also showed the long-range couplings from H-1 to C-1', C-3 and C-10, from H-7 to C-6, C-8, C-9 and C-11, from H-9 to C-1, C-7, C-8 and C-10, from H-11 to C-6, C-7 and C-12, from H-12 to C-7, C-8, C-11 and C-13, from H_3_-13 to C-7, C-11 and C-12, from H_3_-14 to C-4, C-5, C-6 and C-10, from H_3_-15 to C-3, C-4 and C-5. These spectral data, coupling with the following correlations: H-1/H-2/H-3/H-4/H_3_-15; H-12/H-11/H-7/H-6 and H-11/H_3_-13 established by ^1^H,^1^H-COSY correlations (Figure 3), gave rise to another partial structure **1b** (Figure 2). The ester bond linkage, C-1'-O-C-1, between fragments **1a** and **1b** was clearly determined by the HMBC correlation of H-1 with C-1', which permitted the construction of the planar structure of **1** as shown in Figure 2.

The relative configuration of **1** was elucidated by analysis of the partial NOESY data and comparison chemical shifts with berkleasmins A-E and cryptosphaerolide [19,20]. The same relative stereochemistry of C-1, C-4, C-5, C-8, C-9, C-10, C-2', C-3' and C-4' in **1** as in berkleasmins A-E were deduced from the very similar carbon and proton chemical shifts. The β-oriented configuration of H-7 and H_3_-15 was indicated by the observation of NOE interactions (Figure 3) between H-6β (*δ_H_* 1.64 (dd, 13.0, 6.8)) and H-7, and H-6α and H-4, respectively. The relative configuration of H_3_-13 and H_3_-14 should both also be β-oriented deduced from the observation of NOE interactions between H-7 and H_3_-13, and H-6β and H_3_-14, respectively. The *E*-configuration of trisubstituted olefin was assigned by NOESY correlations from H-3' to H-5', and from H-6' to H-14'. Because of some significant signal overlap, we tried to crystallize of **1** in different solvents but finally failed to obtain crystals. Due to small quantity sample, we can not further determine the relative configuration of **1** by chemical methods. Finally, the relative configuration of remaining chiral centers of **1** except for C-6' were determined by comparison chemical shifts with berkleasmins A-E. Unfortunately, the relative configuration of C-6' remains unsigned through only spectroscopic analysis. Through comparison the NMR data of **1** with that of berkleasmin A, the absolute configurations of C-1, C-8, C-2', and C-3' in **1** as berkleasmin A were determined to be 1*R*, 8*S*, 2'*R*, 3'*S*.

Comparison of the physicochemical properties and optical rotation data ([α]_D_^2^^6^ = +10° (c = 0.1, CHCl_3_)) with reported data allowed identifying the compound**2** as berkleasmin C [19], recently reported from the saprobic fungus *Berkleasmium nigroapicale* and shown to possess cytotoxicity against anti-cancer cell-lines (NCI-H187, MCF-7, and KB) and antimalarial activities. The relative and absolute configurations of **2** were from literature [19].

## 3. Experimental

### 3.1. General

Optical rotations: Perkin-Elmer 341 spectropolarimeter. IR spectra: Perkin-Elmer 577 spectrometer; KBr pellets; in cm^-1^. NMR spectra: Bruker AM-600 spectrometer; *δ* in ppm, *J* in Hz; Me_4_Si as internal standard, measured in CDCl_3_. FT-MS spectra: Bruker Apex-Ultra 7.0 T spectrometer, in *m/z*. Column chromatography (CC): silica gel (200~300 mesh, Yantai Zhi Fu chemical Co., Ltd., People’s Republic of China), RP-18 (12 nm, S-50 um, YMC Co., Ltd., Japan), TLC: silica gel GF_254_ plates (Yantai Zhi Fu chemical Co., Ltd, People’s Republic of China) and Sephadex LH-20 gel (25~100 μm, GE Healthcare Co., Ltd., Sweden).

### 3.2. Fungal Material and Cultivation Conditions

*Pestalotiopsis photiniae* was isolated from the branches of *Podocarpus macrophyllus* in Hainan, People’s Republic of China, in April, 2008, and identified by Professor Jing-Ze Zhang, Institute of Biotechnology, Zhejiang University. The isolate was assigned the accession number L328 in the culture collection at College of Life Science, Key Laboratory of Medicinal Chemistry and Molecular Diagnosis of Ministry of Education, Hebei University. The fungal strain was cultured on slants of potato dextrose agar (CPDA ) at 28 °C for 7 days, and then inoculated into a 500 mL Erlenmeyer flask containing 100 mL of medium (glucose 20 g, potato (peeled) 200 g, KH_2_PO_4_ 3 g, MgSO_4_ 1.5 g, citric acid 0.1 g, and thiamin hydrochloride 10 mg in 1.0 liter deionized H_2_O). The final pH of the media was adjusted to 6.5 before sterilization. After 7 days of incubation at 28 °C on rotary shakers at 150 rpm, 25 mL of culture liquid were transferred as seed into each 1,000 mL Erlenmeyer flask containing 250 mL of medium and static fermentation was carried out on a rotary shaker for 30 days.

### 3.3. Extraction and Isolation

The culture broth (20 L) was extracted three times with ethyl acetate. Evaporation of the solvent *in vacuo* gave a brown oily residue (18.0 g), which was subjected to column chromatography (silica gel), eluted with petroleum ether/acetone [100:0, 98:2, 95:5, 90:10, 80:20, 50:50 (v/v)] to afford six fractions *Fr. 1-6*. *Fr. 5* (3.0 g) eluted with petroleum ether/acetone (80:20) was further purified by CC (silica gel; CHCl_3_/acetone, 8:1) to afford eight fractions *Fr. 5.1-5.8. Fr. 5.3* (500 mg) was subjected to Sephadex LH-20 chromatography (CHCl_3_/MeOH, 1:1) to afford compounds **1** (3.0 mg) and **2** (2.5 mg).

*Dihydroberkleasmin* A (**1**): Isolated as white powder, [α]_D_^22^= +70° (c = 0.1, MeOH). IR (KBr) v_max_: 3,424 (OH), 1,736 (C=O), 1,604 (C=C) cm^−1^. ^13^C- (150 MHz, CDCl_3_) and ^1^H-NMR (600 MHz, CDCl_3_): see Table 1. Positive ion ESI-MS *m/z* (%): 545 [M+Na]^+^ (21), 1,068 [2M+Na+H]^+^(7). Positive ion HR-ESI-MS [M+Na]^+^ *m/z* 545.3458 (calcd for C_30_H_50_O_7_Na, 545.3454).

## 4. Conclusions

In summary, we have isolated a new eremophilane-type sesquiterpene, named dihydroberkleasmin A (**1**), together with one known compound, berkleasmin C (**2**), from the culture broth of *Pestalotiopsis photiniae*. Eremophilane-type sesquiterpenes, including those with similar skeletons such as berkleasmins A-C, exist widely as constituents of various plants, while there have been several reports as fungal secondary metabolites mostly from family Xylariaceae. There has been no reported about eremophilane-type sesquiterpenes from the genus *Pestalotiopsis*. 

## References

[1] Suryanarayanan T.S., Senthilarasu G., Muruganandam V. (2000). Endophytic fungi from *Cuscuta reflexa* and its host plants. Fungal Divers..

[2] Toofanee S.B., Dulymamode R. (2002). Fungal endophytes associated with *Cordemoya integrifolia*. Fungal Divers..

[3] Cannon P.F., Simmons C.M. (2002). Diversity and host preference of leaf endophytic fungi in the Iwokrama Forest Reserve, Guyana. Mycologia.

[4] Jeewon R., Liew E.C.Y., Simpson J.A., Hodgkiss I.J., Hyde K.D. (2003). Phylogenetic significance of morphological characters in the taxonomy of Pestalotiopsis species. Mol. Phylogenet. Evol..

[5] Strobel G.A., Hess W.M., Ford E., Sidhu R.S., Yang X. (1996). Endophytic fungi in grasses and woody plants. J. Ind. Microbiol..

[6] Li J.Y., Strobel G.A., Hess W.M., Ford E. (1996). Endophytic taxol-producing fungi from bald cypress. *Taxodium distichum*. Microbiology.

[7] Liu L., Liu S.C., Chen X.L., Guo L.D., Che Y.S. (2009). Pestalofones A-E, bioactive cyclohexanone derivatives from the plant endophytic fungus *Pestalotiopsis fici*. Bioorg. Med. Chem..

[8] Ding G., Li Y., Fu S.B., Liu S.C., Wei J.C., Che Y.S. (2009). Ambuic acid and torreyanic acid derivatives from the endolichenic fungus *Pestalotiopsis sp*. J. Nat. Prod..

[9] Xu J., Kjer J.L., Sendker J., Wray V., Guan H.S., Edrada R., Lin W., Wu J., Proksch P. (2009). Chromones from the endophytic fungus *Pestalotiopsis sp*. isolated from the Chinese mangrove plant *Rhizophora mucronata*. J. Nat. Prod..

[10] Ding G., Zheng Z.H., Liu S.C., Zhang H., Guo L.D., Che Y.S. (2009). Photinides A-F, cytotoxic benzofuranone-derived γ-Lactones from the plant endophytic fungus *Pestalotiopsis photiniae*. J. Nat. Prod..

[11] Liu L., Li Y., Liu S.C., Zheng Z.H., Chen X.L., Zhang H., Guo L.D., Che Y.S. (2009). Chloropestolide A, an antitumor metabolite with an unprecedented spiroketal skeleton from *Pestalotiopsis fici*. Org. Lett..

[12] Liu L., Liu S.C., Niu S.B., Guo L.D., Chen X.L., Che Y.S. (2009). Isoprenylated chromone derivatives from the plant endophytic fungus *Pestalotiopsis fici*. J. Nat. Prod..

[13] Ding G., Jiang L.H., Guo L.D., Chen X.L., Zhang H., Che Y.C. (2008). Pestalazines and pestalamides, bioactive metabolites from the plant pathogenic fungus *Pestalotiopsis theae*. J. Nat. Prod..

[14] Li E., Jiang L.H., Guo L.D., Zhang H., Che Y.S. (2008). Pestalachlorides A-C, antifungal metabolites from the plant endophytic fungus *Pestalotiopsis adusta*. Bioorg. Med. Chem..

[15] Zhang Y.L., Ge H.M., Li F., Song Y.C., Tan R.X. (2008). New phytotoxic metabolites from *Pestalotiopsis sp*. HC02, a fungus residing in chondracris rosee gut. Chem. Biodivers..

[16] Ding G., Liu S.C., Guo L.D., Zhou Y.G., Che Y.S. (2008). Antifungal metabolites from the plant endophytic fungus *Pestalotiopsis foedan*. J. Nat. Prod..

[17] Li E., Tian R.R., Liu S.C., Chen X.L., Guo L.D., Che Y.S. (2008). Pestalotheols A-D, bioactive metabolites from the plant endophytic fungus *Pestalotiopsis theae*. J. Nat. Prod..

[18] Liu L., Liu S.C., Jiang L.H., Chen X.L., Guo L.D., Che Y.S. (2008). Chloropupukeananin, the first chlorinated pupukeanane derivative, and its precursors from *Pestalotiopsis fici*. Org. Lett..

[19] Isaka M., Srisanoh U., Veeranondha S., Choowong W., Lumyong S. (2009). Cytotoxic eremophilane sesquiterpenoids from the saprobic fungus *Berkleasmium nigroapicale* BCC 8220. Tetrahedron.

[20] Oh H., Jensen P.R., Murphy B.T., Fiorilla C., Sullivan J.F., Ramsey T., Fenical W. (2010). Cryptosphaerolide, a cytotoxic mcl-1 inhibitorfroma marine-derived ascomycete relatedtothe genus *Cryptosphaeria*. J. Nat. Prod..

